# Status of Biodiversity in the Baltic Sea

**DOI:** 10.1371/journal.pone.0012467

**Published:** 2010-09-01

**Authors:** Henn Ojaveer, Andres Jaanus, Brian R. MacKenzie, Georg Martin, Sergej Olenin, Teresa Radziejewska, Irena Telesh, Michael L. Zettler, Anastasija Zaiko

**Affiliations:** 1 Estonian Marine Institute, University of Tartu, Pärnu, Estonia; 2 Estonian Marine Institute, University of Tartu, Tallinn, Estonia; 3 National Institute for Aquatic Resources, Technical University of Denmark, Charlottenlund, Denmark; 4 Uni Miljo, Uni Research AS, Bergen, Norway; 5 Coastal Research and Planning Institute, Klaipeda University, Klaipeda, Lithuania; 6 Palaeoceanology Unit, University of Szczecin, Szczecin, Poland; 7 Zoological Institute, Russian Academy of Sciences, Saint Petersburg, Russian Federation; 8 Department of Biology, Leibniz Institute for Baltic Sea Research, Warnemuende, Germany; Institut Pluridisciplinaire Hubert Curien, France

## Abstract

The brackish Baltic Sea hosts species of various origins and environmental tolerances. These immigrated to the sea 10,000 to 15,000 years ago or have been introduced to the area over the relatively recent history of the system. The Baltic Sea has only one known endemic species. While information on some abiotic parameters extends back as long as five centuries and first quantitative snapshot data on biota (on exploited fish populations) originate generally from the same time, international coordination of research began in the early twentieth century. Continuous, annual Baltic Sea-wide long-term datasets on several organism groups (plankton, benthos, fish) are generally available since the mid-1950s. Based on a variety of available data sources (published papers, reports, grey literature, unpublished data), the Baltic Sea, incl. Kattegat, hosts altogether at least 6,065 species, including at least 1,700 phytoplankton, 442 phytobenthos, at least 1,199 zooplankton, at least 569 meiozoobenthos, 1,476 macrozoobenthos, at least 380 vertebrate parasites, about 200 fish, 3 seal, and 83 bird species. In general, but not in all organism groups, high sub-regional total species richness is associated with elevated salinity. Although in comparison with fully marine areas the Baltic Sea supports fewer species, several facets of the system's diversity remain underexplored to this day, such as micro-organisms, foraminiferans, meiobenthos and parasites. In the future, climate change and its interactions with multiple anthropogenic forcings are likely to have major impacts on the Baltic biodiversity.

## Introduction

### Physical and chemical characteristics

The epicontinental and enclosed nontidal Baltic Sea (situated between about 10°–30°E and 54°–66°N) is one of the largest brackish water areas in the world, with a surface area of about 4.2×10^5^ km^2^ and a volume of about 22×10^3^ km^3^, representing about 0.1% and 0.002% of the world's ocean area and volume, respectively. The Baltic Sea is very shallow, with the maximum depth of 460 m and mean depth of 60 m. It was formed after the last glaciation (roughly 10,000–15,000 years ago) and has undergone remarkable shifts in basic physicochemical characteristics during a geologically short time. The contemporary “ecological age” of the Baltic Sea is about 8,000 years ([1] and references therein).

**Figure 1:**
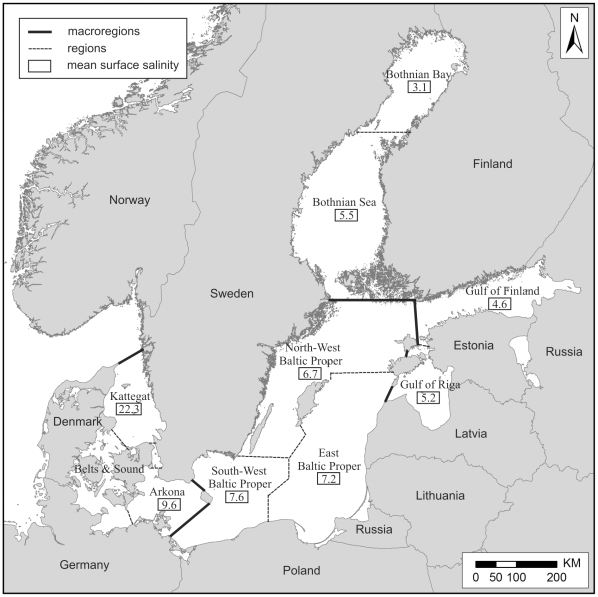
Map of macroregions and regions of the Baltic Sea (sensu [**2**]). Salinity calculated from [195].

**Figure 2:**
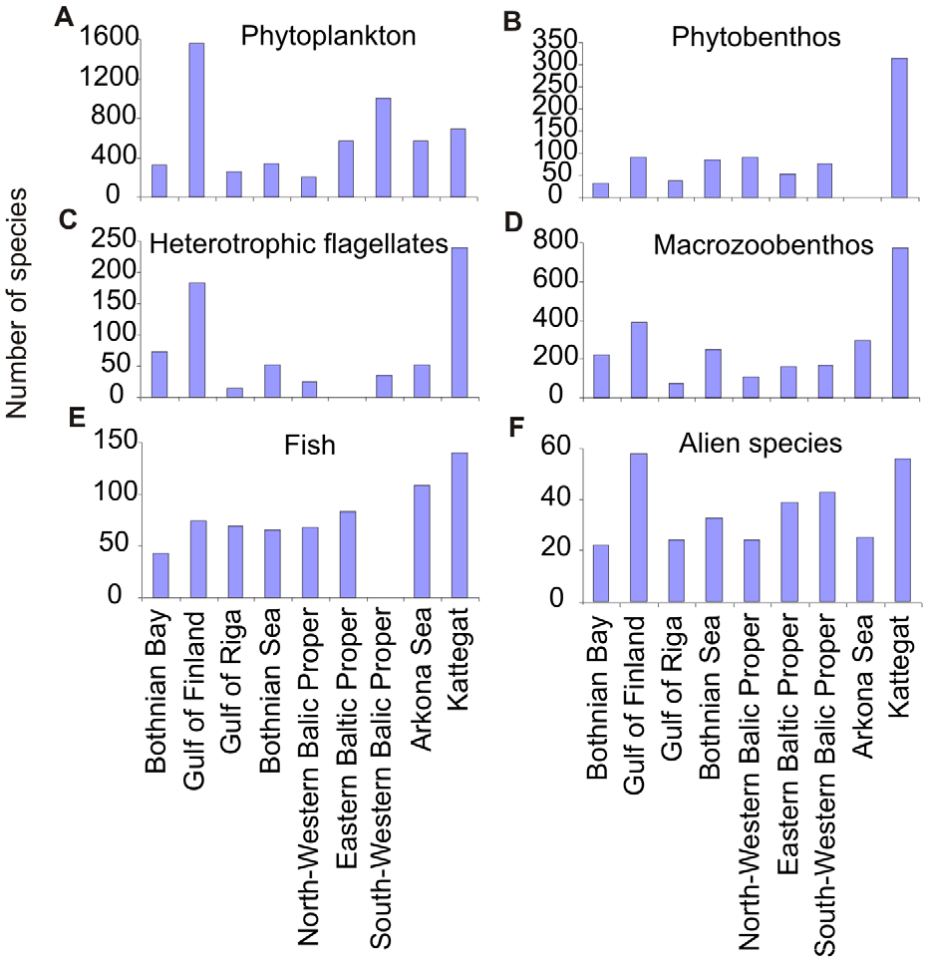
Recorded sub-regional species richness of six organism groups in the Baltic Sea.

The Baltic Sea is composed of 10 regions (Kattegat, Belts and the Sound, Arkona, Southwest, Eastern and Northwest of the Baltic Proper, Gulf of Riga, Gulf of Finland, Bothnian Sea, Bothnian Bay), which could be aggregated into three macrolevel systems—the Transition Area, Baltic Proper, and Large Gulfs (Figure 1, [2]). Nine countries border on the Baltic Sea: Denmark, Finland, Estonia, Germany, Latvia, Lithuania, Poland, Russia, and Sweden. The catchment area is much wider and includes 14 countries with the total area over 1.7×10^6^ km^2^ and about 85 million people [3]. This makes the Baltic vulnerable to a variety of human activities, carried out both *in situ* (pollution, maritime shipping, or fisheries) and on land (e.g., airborne pollutant transfer, nutrient supply via riverine runoff).

The Baltic Sea is situated in the transition area of Atlantic marine and Eurasian continental climate systems, which determines the hydroclimatic conditions of the sea. The most essential are salinity and temperature, both of which have significant gradients, decreasing from southwest to northeast. The salinity regime of the Baltic Sea is determined by two major events: amounts and frequencies of saline water inflows (with high oxygen content) from the North Sea through the Danish Straits and riverine (freshwater) inflows, which are influenced by precipitation [1]. Frequency of major inflows has decreased since the late 1970s. This has caused serious stagnation of the Baltic Sea. The Baltic Sea has a positive water balance: The mean annual freshwater inflow of about 481 km^3^ almost equals the volume of saline water inflows from the North Sea. The major source of freshwater inflow to the Baltic comes via the Gulf of Bothnia, Gulf of Finland, and the Gulf of Riga. The upper water layer is separated from the more saline deepwater layer by a permanent halocline located at depths of about 70–100 m. There is no halocline in the shallower areas in the northeastern Baltic—for example, the Gulf of Bothnia and Gulf of Riga. A strong permanent halocline and seasonal thermocline in summer substantially hamper vertical mixing of water column, which induces formation of oxygen-depleted zones in several locations, essentially in the deep areas of the central Baltic. The water temperature regime is substantially influenced by winter severity. The first sea ice typically forms in November in the Bothnian Bay and remains there until mid-May. Duration of ice coverage decreases from north to south ([4] and references therein). In summer, water temperature in some coastal areas can exceed 25°C. The residence time of the Baltic Sea water is 25–35 years [1].

A notable feature of the Baltic hydrography is the presence of a generally east-west and north-south salinity gradient and salinity stratification of the water column, as already indicated above. The complex hydrographic regime of the Baltic, its short, but dynamic evolution, and the human intervention resulted in the biota consisting of species of various origins and environmental tolerances that have immigrated or been artificially introduced to the area over the relatively recent history of the system. These are marine and freshwater species, migratory species, and glacial relicts. Representatives of these categories have different environmental preferences and the composition of communities therefore varies greatly in different regions of the Baltic Sea, depending primarily on salinity, water temperature, oxygen content, and nutrient concentrations.

### Historical origins of Baltic oceanographic and biodiversity research

Hydrographic measurements were started in the eighteenth century, while measurements at coastal stations and on lightships were initiated in several Baltic countries in the 1890s and regular offshore observations started in the early twentieth century [5]. However, some of the datasets on abiotic parameters (ice breakup dates) extend back as long as five centuries [6]. Several research expeditions studying plankton were carried out in the nineteenth century. The pioneer in the field of modern plankton research, Victor Hensen, developed sampling nets and quantitative methods for studying plankton and used them during expeditions in 1883–86 in the western parts of the Baltic Sea [7]. International coordination of research began in 1902, after establishing the International Council for the Exploration of the Sea (ICES). One of the earliest and most important events was the Russian Baltic Expedition in 1908–1909, during which the first plankton data from several Baltic subareas were obtained (e.g., [8]). While the first information on benthic organisms originated in the eighteenth century, more systematic, although still highly sporadic and mostly qualitative studies on benthos were initiated in the nineteenth century [9], [10]. Synecological analysis of plant and animal communities, together with studies in marine biogeography, characterize the study of Baltic Sea biology during the interwar period (i.e., during the 1920s and the 1930s, [9]). For the fisheries science, the important time baseline is the early 1850s, when K. E. von Baer carried out probably the world's first large-scale study on overfishing of marine fish stocks. However, information on fish and fisheries (such as species descriptions, location of fishing grounds, and conservation measures undertaken) existed before the eighteenth century (e.g., [11], [12] and references therein). Although several regular monitoring cruises were carried out in the 1920s and 1930s, it was only after the mid-1940s, following World War II, that truly systematic research was started, and several long-term continuous datasets on various marine species or taxa have become available since then.

There is at least one major marine biology and fisheries institute or research laboratory in each of the countries surrounding the Baltic Sea. Several of them (including their predecessors) have over 50 years of history. Most of the Baltic countries have several research vessels. The larger vessels include, among others, *Dana* and *Gunnar Thorson* (Denmark), *Maria S. Merian*, *Prof. Albrecht Penck* and *Alkor* (Germany), *Aranda* (Finland), *Vejas* (Lithuania), *Baltica* and *Oceania* (Poland), and *Argos* (Sweden).

The only direct activity of the Census of Marine Life program in the Baltic Sea to create new research network and datasets, and therefore contribute substantially new knowledge, was the History of Marine Animal Populations (HMAP) project. During this project, cooperation between historians and ecologists was initiated and actual research in this interdisciplinary field started. This contributed significantly to the present understanding of the Baltic Sea (especially fish and fisheries) in previous centuries. In addition, Baltic scientists have taken part in other Census projects, such as the Census of Marine Zooplankton (CMarZ), Natural Geography in Shore Areas (NaGISA), Arctic Ocean Diversity (ArcOD), HMAP, History of Nearshore Biodiversity (HNS), Census of the Diversity of Abyssal Marine Life (CeDAMar), and Continental Margin Ecosystems on a Worldwide Scale (COMARGE).

In this study, we summarize the currrent state of knowledge of the biodiversity of the entire Baltic Sea, how this knowledge has progressed through time, and how Baltic biodiversity is influenced by hydrographic conditions, in particular, salinity as well as human impacts. As part of our synthesis, we update species lists and richness data for different organism groups and sub-regions by incorporating new information generated in recent years, based on continued taxonomic monitoring, specific targeted research and application of new molecular genetic methodologies. In addition, we provide original and yet unpublished biodiversity estimates both for relatively well-studied as well as less investigated organism groups. This knowledge should contribute to assessments of how natural and human impacts affect Baltic biodiversity, the development of biodiversity-based indicators of ecosystem status and health, a stronger taxonomic basis for understanding links between biodiversity and ecosystem functioning, and new historical baselines for management of the living marine resources.

## Methods

### Phytoplankton

Substantial samples of phytoplankton from the Baltic Sea have been collected within the framework of national and international monitoring programs. Phytoplankton monitoring in the Baltic Sea is currently to a large extent coordinated through the HELCOM (Helsinki Commission) COMBINE (Cooperative Monitoring in the Baltic Marine Environment) protocol (Text S1). This ensures that the methods of sampling and analysis are similar and that data are comparable. There are differences in the spatial and temporal coverage of samples taken within the different monitoring programs. Most monitoring stations are sampled more frequently during summer. New methods for collecting data, such as ships-of-opportunity and remote sensing, provide additional information to the traditional shipboard sampling.

Changes in methodology constitute the main problem for the comparability of hundred-year-old data with recent data [13]. Some taxa, mainly dinoflagellates and nanoflagellates from different classes, cannot be identified to species or even genus level using routine methods.

### Phytobenthos

The history of hydrobotanical research in the Baltic Sea area dates back centuries, but even now data and comprehensiveness of information about distribution of phytobenthos communities in the area are far from sufficient. Monitoring programs, including a phytobenthos component, exist in all Baltic Sea countries, but most of these programs are targeting not changes of biodiversity itself but effects of human impact, for example eutrophication around known hot spots. Those monitoring programs, techniques used, and indicators or parameters measured are usually country or habitat specific. Few attempts to unify the monitoring methodology have been made (e.g., guidelines developed for HELCOM COMBINE program in 1999), but so far no real intercalibration or harmonization of those techniques has been carried out. Several initiatives are conducted to coordinate the phytobenthos monitoring and mapping methods in the Baltic Sea area in several international programs (e.g., HELCOM Monitoring and Assessment group HELCOM MONAS, Baltic Geographical intercalibration Group Baltic GiG and European Nature Information System EUNIS). Huge effort is currently also directed toward development of efficient and reliable techniques for large-scale spatial mapping of distribution of phytobenthos communities and key species. Use of modern technology, such as underwater video, geographic information system (GIS) modeling, remote sensing, and side-scan sonar is being tested. Large-scale inventory programs have been launched in several countries (e.g., the Finnish inventory programme for the underwater marine environment, VELMU) which all use the results of these new technological developments.

### Zooplankton

The quantity and completeness of published data on zooplankton diversity in different areas of the Baltic Sea vary significantly. In general, biodiversity of estuarine and shallow coastal ecosystems is described better [14] than that of the deep-water basins. In the latter, major zooplankton data originate from the monitoring programs (coordinated by HELCOM COMBINE) that account mainly for the common and most abundant mesozooplankton species (Text S1). Microzooplankters (ciliates and rotifers) of the open Baltic waters are less studied so far, although their contribution to the total zooplankton diversity is the greatest. However, most often difficulties in treating the samples and lack of specialists capable of adequate species identification hamper incorporation of biodiversity investigations into the routine monitoring programs.

Illustrated atlases with drawings and photos of live and preserved planktonic organisms are available for the Baltic Sea. A recent series of zooplankton atlases of the Baltic Sea [15]–[17] and the references therein present species descriptions, line drawings and plentiful photo illustrations, methodology of zooplankton studies in the shallow coastal and deep-open Baltic waters, the sampling strategy, periodicity and intensity, sampling gear for all zooplankton size classes, methods of treating the samples and data analyses (species identification, counts, biomass determination, etc.), and the relevant bibliography.

### Meiozoobenthos

Meiozoobenthos is operationally defined as benthic invertebrates that pass through the sieves with 0.5 mm (or 1 mm) mesh size and are retained on those with 0.044 mm (or 0.063 mm) meshes [18]. In addition to being defined operationally, the meiozoobenthos is currently regarded as a valid ecological category among the marine benthos in part because of its being distinctly separated in benthic biomass size spectra [19], [20]. Although including larger protists (particularly foraminiferans and ciliates), the size class/ecological category in question is usually studied with the focus on metazoan invertebrates. Identification aids that specifically deal with the Baltic meiozoobenthos are rare or nonexistent (Text S1); therefore, researchers have to rely on broader-scope works that subsequently will have to be supplemented by reference to the available databases, such as NeMys for nematodes (http://nemys.ugent.be) or detailed taxonomic publications. The current overview is based on data contained in the European Register of Marine Species (www.marbef.org) and on a literature survey and unpublished information. The major meiozoobenthic phyla are discussed in this paper in a taxonomic sequence consistent with that of Higgins and Thiel [21].

### Macrozoobenthos

Numerous literature sources were analyzed for information on macrozoobenthos distribution in the Baltic Sea. Other relevant information was captured from the IfAÖ Autecological Atlas [22], HELCOM monitoring data, and the Baltic Sea Alien Species Database. During the past 30 years, the Leibniz Institute for Baltic Sea Research (IOW) sampled several regions and regular observational stations both as part of the regular monitoring and within different research programs.

All macrofauna species (taxon units) were identified to the lowest taxonomic level possible. The nomenclature was checked following the European Register of Marine Species (http://www.marbef.org/data/erms.php). The taxonomic nomenclature used in the historical studies was revised before including the species in the analysis. For some species or data entries, the taxonomic assignment was highly doubtful, or not possible, when following present-day taxonomical concepts. In these cases the data were excluded. Some difficult taxonomic groups (e.g., hydrozoans, turbellarians, nemertines, bryozoans, sponges, and oligochaetes) are likely to be underrepresented because of the different expertises of the authors. At the same time, special consideration is devoted to freshwater species, including those found in the brackish and freshwater bodies adjacent to the Baltic Sea.

Revised data on species occurrence within the defined Baltic Sea subregions were compiled in GIS (software ArcGIS 9.1, ESRI, USA). Unique ranges of taxon units were filtered out for each of the defined subregions.

### Fish

Monitoring of the commercially most important species (cod, herring, sprat, salmon, and sea trout) is carried out in all Baltic countries according to guidelines and procedures agreed in forums such as the International Council for the Exploration of the Sea (ICES) and the European Union (Text S1). The monitoring is based on a combination of research surveys and commercial catch information for cod, herring, and sprat, and catch and effort information for salmon and sea trout [23]. Abundance of each species is estimated for different areas of the Baltic (e.g., Gulf of Bothnia, Gulf of Riga, central Baltic Sea, Kattegat), which correspond partly to regional differences in both the ecology (e.g., growth, reproduction, and migration) and the fisheries for different species and populations. The surveys collect many other species as bycatch, so they can potentially be used to monitor changes in the overall fish community and biodiversity (e.g., changes due to fishing, species introductions, or climate change). Hydrographic data are also collected at each sampling station on the surveys. Identifications are made using local fish taxonomic and atlas guides (e.g., [24]).

Information on all other fish is being obtained within coastal fish monitoring programs. These are in place in all Baltic countries, covering different parts of the Baltic Sea and various environmental conditions. The time series cover up to 22 years of annual monitoring. Fish sampling methodology is elaborated in detail and generally unified across the countries [25], [26]. Fish catches at each station are registered in numbers per species, separated in centimeter length groups and mesh sizes. Weather conditions and some key hydrographic parameters are measured as well [25]. As the coastal fish monitoring is mainly directed toward demersal and benthopelagic species during the warm season, small-sized species such as sticklebacks (Gasterosteidae), gobies (Gobiidae), and pipefish (*Nerophis ophidion*) are rarely caught, as are the cold-water preferring glacial relict species [26].

### Historical studies

The biodiversity- and ecosystem-related research activities have generally followed a three-step plan, as identified and developed in global and Baltic HMAP workshops [27]–[29]: (1) Identify ecological hypotheses related to long-term variations in abundance and catches of fish and marine mammals; (2) Develop national overviews of the available materials and sources for all Baltic countries, identifying which materials could potentially be the most useful for addressing HMAP objectives and should be investigated in detail; (3) Based on the established knowledge of the archival deposits, develop or modify hypotheses, select and undertake studies of the historical sources, and evaluate hypotheses using historical data generated during the HMAP project.

In addition, an important initial task was to create a dialogue among historians, archaeologists, paleoecologists, and fisheries and marine mammal ecologists. The main sources of information that were considered as potentially useful for addressing Baltic-HMAP objectives include (1) Quantitative sources such as annual tax accounts, customs rolls, household accounts, commercial catches, and scientific materials on the Baltic ecosystem and its fish populations; (2) Qualitative sources including fishing commissions' records, reports from government officers, public grants records, private sources and topographical literature; (3) Archaeological documentation, which consists mainly of subfossil fish bones and evidence of fluctuations in the productivity of the marine biotope.

Baltic HMAP covers a broad range of species and populations of different origins characteristic for the Baltic Sea (marine, freshwater, migratory, and glacial relict species); these species have different life history traits and environmental preferences in as many subsystems as possible. However, during the course of the project, the limitations of the availability of historical data became evident, and consequently, the main focus narrowed to only a few major marine species, for which the most extensive data could be recovered with the available resources. These species were cod (essentially the eastern Baltic cod population) and herring, which have several distinct populations in the Baltic Sea.

Quantitative approaches, including standard stock assessment models were applied for extending the knowledge of stock dynamics of the eastern Baltic cod in the twentieth century [30], [31]. For the other time periods and species, the new information provided was mainly related to catches and developments in fisheries, which in some situations are able to indicate qualitative developments in the fish stocks.

### Bioinvasions

The main source of information on nonindigenous species in the Baltic Sea area is the Baltic Sea Alien Species Database [32], which was essentially updated in the course of DAISIE (Delivering Alien Invasive Species Inventories for Europe) [33], a recent project funded by the EU Framework Program. In addition, yet unpublished data and other information was used. An alien species (synonyms: nonnative, nonindigenous, exotic, introduced) was defined as a species intentionally or unintentionally introduced by humans outside its past or present natural range and dispersal potential ([34]; for recent reviews of alien species terminology see, for example, [35]). Natural shifts in distribution range, such as those due to climate change or dispersal by ocean currents, do not qualify a species as an alien. Also it is important to distinguish between alien and invasive species. The latter is defined as an alien species for which “population has undergone an exponential growth stage and is rapidly extending its range” [35] or its “introduction does, or is likely to, cause economic or environmental harm or harm to human health” [34].

## Results

Based on a variety of different source material (i.e., journal articles, published reports, grey literature, unpublished data), the documented total number of cyanobacterial, phytoplankton, zooplankton, phytobenthos, zoobenthos, fish, marine mammal, and bird species as well as vertebrate parasites inhabiting the Baltic Sea is at least 6,065. Importantly, this estimate contains several yet unpublished sources for several both relatively poorly and well-studied organisms groups (for instance, parasites of vertebrates and macrozoobenthos, respectively). However, the real estimate is most likely substantially higher as the current knowldege-level on several taxa and/or organism groups (incl. foraminiferans, micro-organisms, meiobenthos, parasites) appears to be relatively incomplete. Moreover, the estimated number of species of heterotrophic bacteria could be as high as up to 10^6^ (see also Table 1 and Table 2). The following sections provide detailed information on species diversity, distribution and abundance patterns by the major organism groups, and also describe and analyse some characteristic and important issues with respect to a given organism group, e.g., the treatment of meiobenthic major taxa or stock status and applied aspects of commercial fish biodiversity. In addition, emphasis is given to historical aspects of research as well as to listing important publication sources.

**Table 1:** Estimated number of species, laboratories and scientists by major organism groups in the Baltic Sea.

	Number of species	Number of laboratories	Number of scientists
Bacteria	10^3^–10^6^ a	10	50
Phytoplankton	1,700 b	15	50
Phytobenthos	442 c	15	60
Zooplankton	1,199 d	30	50
Meiozoobenthos	569 d	<5	<10
Macrozoobenthos	1,476 d	13	15
Parasites of vertebrates	380 e	10	15
Fish	200 f	15–20	150
Seals	3 g	14	25
Seabirds	83 h	?	?

**Sources**:

a Estimate: H. Kaartakallio, unpublished.

b [36].

c [51].

d This study.

e [135], [156]–[158]. Includes also: A. Turovski, unpublished data.

f [54], [134]–[136]. Includes lampreys.

g [54].

h C. Herrmann, unpublished. Includes species which have special relation with the Baltic marine environment (breeding, migration, wintering).

**Table 2:** Taxonomic classification of species reported in the Baltic Sea area, incl. Kattegat.

Taxonomic group	Estimated no. of species^1^	State of knowledge^2^	No. of alien species^3^	No. ID guides^4^
**Domain Archaea**	**?**	**1**	**?**	**-**
**Domain Bacteria (including Cyanobacteria)**	**? (200)** 5	**1 (5)**	**? (0)**	- (4)
**Domain Eukarya**				
**Kingdom Chromista**	**963**	**4**	**7**	4,9
Phaeophyta	130	4	3	4, 9
**Kingdom Plantae**				
Chlorophyta	505	4	2	4, 18, 20
Rhodophyta	150	4	4	18
Angiospermae	20	4	1	16
**Kingdom Protoctista (Protozoa)**	**963**	**3**	**2**	
Dinomastigota (Dinoflagellata)	88	4	2	77
Foraminifera	96	2	0	
**Kingdom Animalia**				
Porifera	25	4	0	77
Cnidaria	99	4	5	31, 40, 67, 77
Platyhelminthes	313	3	2	58, 77, 82–87, 90
Mollusca	318	5	12	75, 77, 76, 81
Annelida	388	4	12	50, 52, 76
Crustacea	607	4	33	57, 61, 73, 51, 53–54, 22–27, 36, 37, 41, 42, 45, 46, 77, 79, 80, 82–87
Bryozoa	68	4	1	77
Echinodermata	52	5	0	77
Urochordata (Tunicata)	26	4	1	38–40, 44, 46–48, 77
Other invertebrates	765	3	3	62–63, 72, 56, 59–60, 66, 70, 64, 69, 65, 49, 71, 47, 48, 77, 82–87, 90–91
Vertebrata (Pisces)	200	5	29	92, 93, 97, 98, 100
Other vertebrates	89	4	3	101–106
**SUBTOTAL**				
**TOTAL REGIONAL DIVERSITY** 3	**6,065**		**117**	

1 Estimated number of species in data sources from Table 1 and [194].

2 State of knowledge: 5 = very well-known; 4 = well-known; 3 = poorly known; 2 = very poorly known; 1 = unknown.

3 Number of alien species from [32].

4 Identification guides cited in Text S1.

5 Numbers in brackets indicate information for cyanobacteria.

### Phytoplankton

Knowledge of the taxonomy and distribution of Baltic Sea phytoplankton has increased considerably over the past three decades. The *Checklist of Baltic Sea Phytoplankton Species* comprises over 1,700 recorded species [36]. An extensive examination of plankton from the Kattegat area listed about 400 species of planktonic algae and heterotrophic flagellates [37]. Because of the various taxonomic research levels in different sub-basins of the Baltic Sea, many species apparently have a much wider distribution than the records in the checklists indicate.

By the identified sub-regions, phytoplankton species diversity is the highest (1,565 species) in the Gulf of Finland and the overall sub-regional biodiversity at low salinity conditions (below 10) is about 13% lower than that in the high salinity conditions in Kattegat (692 species, Figure 2A). Diatoms and dinoflagellates are characteristic in the saline waters of the southern Baltic Sea, the Belt Sea, and the Kattegat, whereas phytoplankton groups preferring less saline water, such as cyanobacteria and chlorophytes, are commonly found in the northern Baltic Sea, where low water temperature and winter ice cover also influence the phytoplankton community and timing of events, such as onset of the spring bloom [38], [39]. Cyanobacteria usually dominate in the coastal and open areas of most sub-basins of the Baltic Sea in summer, with the exception of the Belt Sea, the Kattegat and the Gulf of Bothnia (e.g., [40]–[42]).

A list of potentially harmful phytoplankton in the Baltic Sea contains over 60 species with effects connected to toxicity, mechanical disturbance, bloom formation and water coloration [43]. Recent blooms of the potentially toxic dinoflagellate *Alexandrium ostenfeldii* have been recorded in the Gulf of Gdańsk and the Swedish east coast of the northern Baltic Proper causing bioluminescent events [44].

It has been proposed that many recent changes in the phytoplankton could be related to climate variation, which influence directly and indirectly water temperature, salinity, and loading from the catchment in the Baltic Sea area [42], [45].

Analysis of historical and present day phytoplankton composition data shows that many phytoplankton taxa are now more frequent, and their seasonal dynamics have changed, since the early 1900s [13], [46]. In addition to cyanobacteria (e.g., [46]), long-term records provide evidence that the biomass of chrysophytes and chlorophytes in the surface water has increased significantly in the open northern Baltic Sea [47]. In coastal waters the shifts in phytoplankton composition are typically not abrupt and the changes are rather small if the increases in nutrient levels are small or moderate [48].

While clear trends are largely missing from the last decades, the reported long-term increases in cyanobacteria and the blooms of invasive species indicate that the Baltic Sea phytoplankton is not at its “natural level” as targeted in the HELCOM Baltic Sea Action Plan (BSAP) [49]. At present enhanced internal loading of phosphorus and the removal of dissolved inorganic nitrogen leads to lower nitrogen-to-phosphorus ratios, which favor blooms of nitrogen-fixing cyanobacteria [50]. This complicates the target for short-term reduction of present blooms. The establishment of new species and changes in the species composition indicate changes in phytoplankton biodiversity. The reasons behind the biodiversity alterations are not fully understood. Also, the effects of the biodiversity modification on the Baltic Sea ecosystem cannot yet be determined. The changing climate, with associated higher probability of extreme weather events, is likely to increase the risk for new species introductions and unexpected blooms in the Baltic Sea area.

### Phytobenthos

At the present time, 442 species of macroalgae [51] are recorded in the Baltic Sea including Kategat area. As is typical for most brackish water systems, the number of marine phytobenthic species decreases with the salinity gradient, as salinity is the main environmental factor controlling the wide-scale distribution of species on the Baltic Sea, while exposure, substratum type, and light availability determine the structure of vegetation communities on the local scale. In general, the pattern is of a decline in the number of species belonging to the Bangiophyceae and Fucophyceae and an increase in the Chlorophyceae along the falling salinity gradient [51].

While the total number of phytobenthic species is rather high for the whole Baltic Sea area, the sub-regional diversity is often much lower (Figure 2B). For instance, for the Gulf of Riga, the total number of macroscopic phytobenthos species is 39, including 12 species of aquatic higher plants [10]; for Gulf of Finland the total number of macroscopic algae is 91; and for the northernmost part of the Baltic Sea—Bothnian Bay—the number is 33 [51].

The Baltic Sea has one known endemic phytobenthic species – *Fucus radicans* [52]. At the same time there are eight species of macroalgae and four species of vascular plants listed in the *HELCOM Lists of Threatened and/or Declining Species and Biotopes/Habitats in the Baltic Sea Area* [53]. Among those are species associated with very specific habitats, such as charophytes, but also species that suffer from large-scale environmental problems of the Baltic, such as eutrophication effects (e.g. two key species found in hard-bottom habitats *Fucus vesiculosus* and *Furcellaria lumbricalis*).

Only a small number of species have significant roles in the ecosystem, providing, along with their physical structure or physiological performance, the necessary environmental support for other species. These species are able to modify the environment physically and structure the habitat to provide suitable conditions for a large number of species. In the Baltic Sea such special, structuring species are usually large perennial macroalgae on hard bottoms and phanerogams and charophytes on soft bottoms.

Recent trends in the phytobenthos biodiversity are described in the latest report published by HELCOM [54]. The largest concern is decline of distribution areas of key species of both hard (*Fucus vesiculosus*, *Furcellaria lumbricalis*) and soft (*Zostera marina*, charophytes) bottoms. The decline of those communities has been described as a long-term process with duration of decades [55], [56]. In some limited areas in the northeastern Baltic Sea (such as Stockholm Archipelago and Tallinn Bay), this process has been reversed during recent years, indicating improvement of the eutrophication situation in some coastal areas [54].

### Zooplankton

The overall species richness of micro- (20–200 µm), meso- (0.2–20.0 mm), and macrozooplankton (larger than 20 mm) in the whole Baltic Sea is 1199 species with open Baltic hosting 1031 and estuaries 168 species [15], [16], [57]. Results of the recent zooplankton revisions are at variance with former assessments based on insufficient biodiversity knowledge [58]–[61] and, consequently, with the outdated affirmation that “the number of species in the Baltic is low” [62]. The newest zooplankton inventories [17], [57] illustrate the diversity of the open Baltic Sea and present a checklist of 814 species of protozooplankton (Ciliophora) and 217 species of metazooplankton organisms: Cnidaria, Ctenophora, Turbellaria, Rotifera, Phyllopoda, Copepoda, Chaetognatha, and Copelata, as well as meroplanktonic larvae of Polychaeta, Mollusca, Cirripedia, Bryozoa, and Echinodermata. Nearly 400 species of planktonic ciliates, rotifers, and crustaceans are known from major estuarine and coastal ecosystems of the Baltic Sea [15], [16], [63]. If the heterotrophic nanoflagellates are considered [36], the total zooplankton diversity in the Baltic Sea increases significantly (see section “Phytoplankton”).

The sub-regional diversity of heterotrophic plankton may be best illustrated by flagellates (Figure 2C). The highest species richness is registered in Kattegat (240 species) while the lowest in the Gulf of Riga. Although low values of heterotrophic flagellates' diversity in several sub-regions may witness for the insufficient taxonomic knowledge rather than low biodiversity of the group [64], the general results still indicate that relatively the highest diversity is present in high-salinity conditions. Similarly, detailed taxonomic data on some other and more abundant zooplankton groups is lacking in a number of sub-regions of the Baltic Sea [57].

According to present-day knowledge, the most species-rich component of the Baltic Sea zooplankton is microplankton (ciliates and rotifers). The greatest overall diversity was registered in ciliates, among which 166 species are holoplanktonic, while about 650 benthopelagic species inhabit presumably the near-bottom layers and shallow waters [17], [57], [64]. Ciliates contribute roughly 70% to the total zooplankton species richness. The dominant groups are small aloricate Oligotrichida (genera *Strombidium, Strobilidium, Lohmaniella*) and tintinnids – ciliates with lorica (e.g., [65]–[67]). Hymenostomatida (mainly small scuticociliates *Cyclidium, Cristigera, Balanion*) and Litostomatea (*Mesodinium, Didinium, Monodinium*) are also rather abundant [64]. Some of these groups are also dominant in the Baltic Sea ice [68]. The taxonomic diversity of the smaller zooplankton fraction (ciliates with body length below 20 µm and heterotrophic flagellates) is still in need of revision [64].

**Figure 3:**
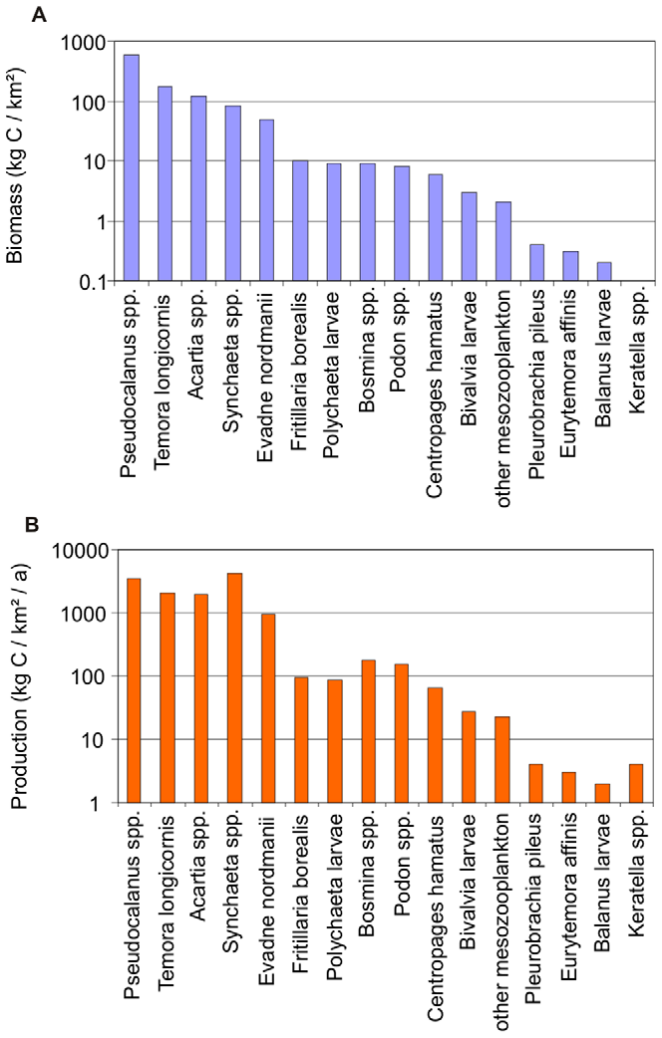
Annual zooplankton (A) biomass and (B) production by taxa in Gdansk Bay in the 1980s (modified from [**196**]).

Rotifers are responsible for 15% of the total Baltic zooplankton species richness; they are especially diverse and abundant (up to 95% of zooplankton biomass) in the coastal ecosystems (e.g., [69]). Rotifers decrease in diversity and in numbers with increasing water salinity, due to the freshwater origin of this group. The most species-rich rotifer families in the Baltic Sea are Synchaetidae (*Synchaeta* spp., *Polyarthra* spp.) and Brachionidae (*Brachionus* spp., *Keratella* spp.). These rotifers contribute significantly to the total zooplankton biomass and production, also in the open Baltic waters (Figure 3A,B).

Within the meso- and macrozooplankton, copepods *Pseudocalanus* spp., *Temora longicornis*, *Acartia* spp., and cladocerans *Evadne nordmanni* are the most important taxa in the open Baltic in biomass and production. Ctenophores *Pleurobrachia pileus* and copepods *Eurytemora affinis* play a minor role, while appendicularians *Fritillaria borealis*, Polychaeta larvae, cladocerans *Bosmina* spp., *Podon* spp., copepods *Centropages hamatus* and Bivalvia larvae range in between (Figure 3A,B).

There are about 40 mesozooplankton species that regularly occur in the open Baltic Sea with high abundance, 10–12 of which are dominating taxa [17]. Semiquantitative description of the presence of the dominant taxa in the Baltic Sea regions from Kattegat to the Gulf of Finland and Bothnian Bay [70] allowed ranking the occurrence of various zooplankters, revealing a remarkable shift in the dominating taxonomic groups from west to northeast (Table 3). Partly in the eastern Kattegat and especially in the Sound, the zooplankton species composition demonstrates similarities to that in the near-surface waters of the Arkona Sea, for example, by the occurrence of *Acartia* species, which is a result of the Baltic Sea water outflow. Meanwhile, copepods *Acartia bifilosa*, which tolerate salinity of 0.30 [71], and *Eurytemora affinis*, which survive at salinity of 0.50 [72] are the key species in the Gulf of Finland and the Bothnian Sea. Finally, a two-layer distribution of zooplankton in the Bothnian Bay was described [70]: while the glacial relict copepod *Limnocalanus macrurus* inhabits the cooler brackish deep waters, *Daphnia* species are distributed in the nearly freshwater surface layers. *C. hamatus* is a subdominant in the Baltic; occurring at maximum population densities from Kattegat to the Arkona Sea. The Baltic Proper is the area where *Acartia* species, *T. longicornis*, and *Bosmina* spp. (in summer) dominate.

**Table 3:** Sub-regional dominance shift of the most common zooplankton taxa in the Baltic Sea across the salinity gradient from A to K.

Taxa/Subregions	A	B	C	D	E	F	G	H	I	J	K
*Paracalanus parvus*	1	2		5^a^							
*Pseudocalanus* spp.	2	1	1	2	2	2	1	1	4	3	
*Oithona similis*	3	4	4	1	4						
*Centropages hamatus*	4	3	2	3	3	4					
Carnivorous cladocerans^b^	5	5			5^c^						
Meroplanktonic larvae	6			4^d^							
*Calanus finmarchicus*	7										
*Centropages typicus*	7										
*Acartia* spp.		6	3	4	1	1	2	2			
*Oikopleura dioica*			5^e^								
*Temora longicornis*						3	3	3			
*Bosmina* spp.						5^d^	4^d^	4^d^	3^d^	2^d^	
*Evadne nordmanni*							5				
*Acartia tonsa*				5^a^							
*Acartia bifilosa*									1	1	
*Eurytemora affinis*									2	1	
*Limnocalanus macrurus*									4	3	1
*Synchaeta* spp.									5^f^	4^f^	
*Fritillaria borealis*									6		
*Pleurobrachia pileus*									6		
Polychaeta (larvae)									6		
*Keratella* spp.										4^f^	
*Daphnia* spp.											2

**Note**:

Modified from: [185].

**Legend**:

1– the lowest, 7– the highest.

A Kattegat (shallow areas).

B Kattegat (deep areas).

C The Belts.

D Kiel Bay.

E The Sound.

F Arkona Sea.

G Bornholm Sea.

H Gotland Sea.

I Gulf of Finland.

J Bothnian Sea.

K Bothnian Bay.

a late summer/autumn.

b *Evadne nordmanni, Podon* spp.

c not numerous.

d not every year.

e in spring.

Long-term variability in the atmospheric and, consequently, hydrographic regime also causes alterations in mesozooplankton abundance and in species composition, of which the main driving forces are shifts in salinity and temperature. For example, the prolonged period of missing saltwater inflows and increasing river runoff in the Northern Baltic and the Gulf of Finland during the late 1980s caused the penetration of eight *Keratella* species and several other rotifers (*Polyarthra* spp., *Kellicotia longispina*), as well as the cladocerans *Bythotrepes longimanus* into the Northern Baltic Proper [73]. At the same time, the key species changed in the Central Baltic Proper: the formerly dominating halophilic representatives of the cold-water genus *Pseudocalanus* were substituted by *Acartia* species, while in the northern parts of the Baltic Proper, the dominance of *Acartia* spp. was replaced by the brackish-water *E. affinis* (e.g., [74]–[76]).

### Meiozoobenthos

As already said, the discussion of meiozoobenthos in this paper focuses on metazoan invertebrates, hence it disregards protists (i.a., foraminiferans and ciliates, the latter known to be abundant and the former occasionally speciose in the Baltic sediments). At least 569 species have been identified in the Baltic meiobenthic communities, although the actual number is probably much higher (see below). In the Baltic Sea, meiobenthos is quantitatively prominent, particularly in areas below the halocline where the abundance and biomass of the macrobenthos decline sharply because of hypoxia and anoxia (e.g., [77]). On the anoxic bottom of the Baltic Proper, it is only members of the meiobenthos that are able to withstand the stress exerted by the lack of oxygen [78], [79]. Owing to salinity constraints in the Baltic, numerous major marine meiobenthic taxa are either totally absent (e.g., Loricifera, Gnathostomulida) or their distribution is confined to the western, southwestern, and southern part of the Baltic. The following gives a short overview by major taxa:

#### Cnidaria

The phylum Cnidaria is represented in the Baltic Sea by three species: *Halammohydra octopodides*, so far found living in coastal sandy sediments off the southwestern and southern Baltic coast [80], *Protohydra leuckarti* [81], and *Chlorohydra viridissima*, present in the Gulf of Riga [82].

#### Turbellaria

Turbellarians are a common platyhelminth grouping in the Baltic meiobenthos, particularly in sublittoral sandy bottoms off the southern Baltic coast (e.g., [79], [83]; Radziejewska, unpubl. data) and on the southern Baltic beaches (e.g., [84]), where they occasionally dominate. The most thorough account of Baltic turbellarians to date [85] provides a list of 134 species. Because the identification of turbellarians requires examination of live material, the qualitative aspect of turbellarian taxocene is, as a rule, ignored in routine studies.

#### Nematoda

The nematodes are ubiquitous members of the meiobenthos and dominate in meiofaunal assemblages in most benthic habitats, irrespective of sediment type, depth, and oxygen situation [18]. They are the only metazoans not eliminated by oxygen deficiency (e.g., [79]. Nematodes are probably the most diverse metazoan meiobenthic taxon in the Baltic as well, although no overall list of the Baltic species has been compiled so far. The existing area-specific publications refer predominantly to the number of genera [86], [87], or report lists of genera and working or putative species [88]. Based on the available information, it may be expected that the list of nematode species in the Baltic should feature at least 200 species.

#### Gastrotricha

Knowledge on the species richness of the Baltic gastrotrichs derives from only two publications [89], [90] and the identification of 33 species. The total number of gastrotrich species is brought to 34 by *Musellifer profundus*, a species found so far at a few deep localities in the Baltic Proper [91], but which may also occur in much higher density in the western Baltic.

#### Rotifera

In marine habitats, rotifers are usually associated with coastal sandy sediments. However, knowledge of rotifers occurring in the Baltic is scanty. The major accounts of rotifer species richness in the Baltic were provided in the 1940s through the 1960s [92], [93] with a total list of 34 species. Recently, two additional rotifers were described in the western Baltic [94].

#### Kinorhyncha

An exclusively meiobenthic phylum, kinorhynchs are fairly frequently encountered in deeper parts of the Baltic Proper [79] and relatively abundant in the western Baltic, but have not been taxonomically identified in most of the Baltic meiobenthic surveys to date. So far, only two species have been described: *Echinoderes levanderi* [85] and *Pycnophyes maximus* [95].

#### Oligochaeta

As no special study of meiobenthic oligochaetes has been carried out in the Baltic, it is highly probable that meiobenthic-sized representatives of the taxon are listed together with their macrobenthic counterparts. So far, lists of representatives of those families in the Baltic Sea contain about 20 species [81], [82], [96].

#### Tardigrada

Aquatic representatives of the meiobenthic phylum Tardigrada are commonly encountered in the Baltic in sandy habitats, both littoral (beaches) and sublittoral. So far, three known species have been reported [97].

#### Ostracoda

The total number of ostracod species recorded in the Baltic is 40, making those crustaceans one of the most species-rich taxa among the meiobenthos. Representatives of this taxon are found in various benthic habitats, except for deeper areas stressed by oxygen deficiency. In some parts of the Baltic, ostracods may account for most of the total meiobenthic biomass, constitute an important link in pelagic-benthic couplings, and are efficient bioturbators [98]. However, biodiversity-related information on Baltic ostracods derives primarily from studies in different coastal areas [81], [82], [96], [99].

#### Harpacticoida

Because of the intensity of the research effort, the total number of harpacticoid species in the Baltic is quite high and totals 82. Harpacticoid assemblages have been identified in many areas and have been relatively well studied, particularly in the southern Baltic [100]–[106]. These animals occur in most of the Baltic benthic habitats, except for the bottoms stressed by oxygen deficiency.

#### Halacaroidea

Halacaroid species are exclusively meiobenthic acarids that are fairly common in sandy sublittoral areas of the Baltic [83], particularly where the sediment is enriched with algal remains [107]. A total of 14 species have been recorded in the Baltic [108], although it is probable that the list is far from complete [18].

### Macrozoobenthos

Regular study of macrozoobenthos in Baltic waters dates back to the end of the nineteenth century. The systematic observation of species and their distributions in the Baltic Sea began with Möbius, who published two papers on invertebrates in 1873 and 1884 [109], [110]. Other famous scientists of this time period were Lenz, Brandt, and Nordquist [111], [112]. Many other papers exist from this time period, but in most cases they only dealt marginally with macrozoobenthic species, or were mainly motivated by fisheries-related questions. Especially at the beginning of the twentieth century, the famous research cruises [113]–[126] were focused on macrozoobenthos and food of commercial fishes of the Baltic Sea. The results of this research could serve as *status quo ante* for comparative studies in recent times. Particularly in respect to the EU Directives for Water Framework and Marine Strategy, a baseline is needed for the definition of pristine areas and their macrozoobenthic communities. Since the mid-1950s, scientific efforts investigating the benthic fauna of the Baltic Sea have increased rapidly, resulting in the publication of several hundred papers. A comprehensive overview of historical and current literature on macrozoobenthos is given for the German Baltic area by Gerlach, Zettler, and Röhner [127], [128].

In brackish water systems such as the Baltic Sea two main environmental variables (salinity and oxygen supply) affect the composition of the benthic community and species' abundance (e.g., [129]). Within a few hundred kilometers to the east or the north, the salinity values decrease from about 30 down to 5 and, finally, in the most northern part to more or less freshwater conditions. As a consequence, the number of marine species is significantly decreased or has been displaced by limnic species in the north and inner coastal waters [130], [131]. Oxygen availability also limits species' distribution because most benthic organisms are sensitive to long-term low-oxygen conditions [132]. Therefore, benthic life is often absent in the deeper basins below the halocline particularly after longer periods without saline water inflows. Even though the Baltic is a comparatively young ecosystem which is species-poor and vulnerable to the threat of invasive marine and exotic species, both the strong gradient and the rapid change in salinity conditions especially in the southern Baltic inhibit an unhindered colonization. As a result, the Baltic benthic fauna is still largely characterized by species with obviously opportunistic life history traits [133] and the total macrozoobenthic species diversity is an average, 3.7 times lower than in sub-regions (e.g., Kattegat containing 775 species) characterised by low-salinity conditions (below 10) (Figure 2D).

**Figure 4:**
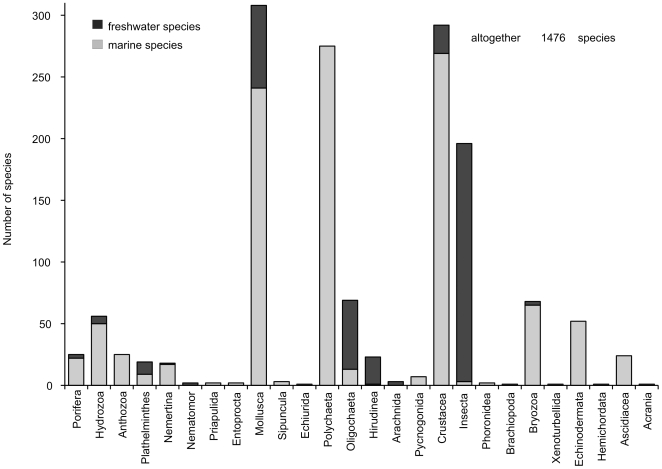
Recorded macrozoobenthos taxonomic composition in the Baltic Sea, based on historical and recent data. The spatial compoment is given on Figure 5.

**Figure 5:**
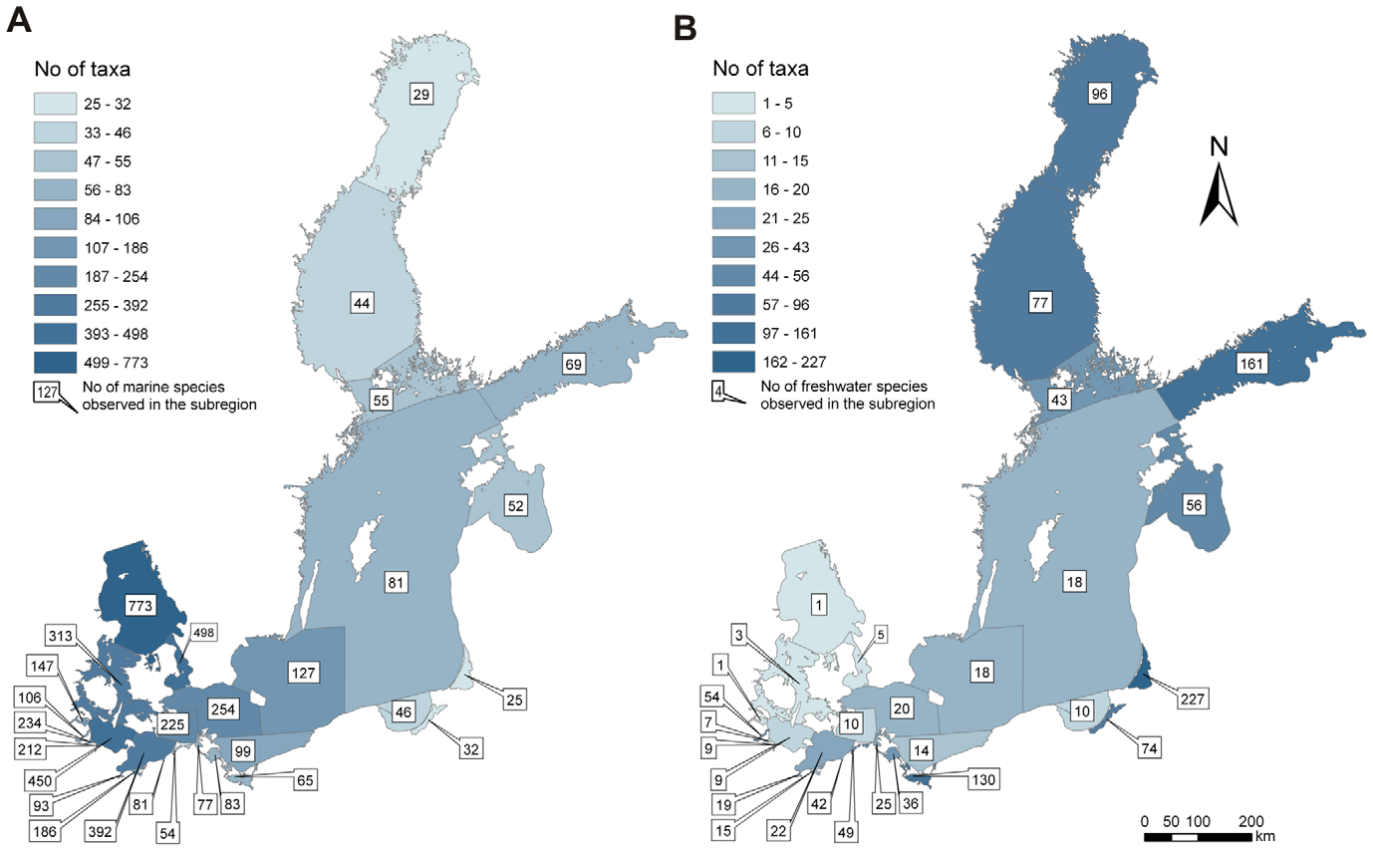
Sub-regional distribution of (A) marine and (B) freshwater taxa in the Baltic Sea: case of macrozoobenthos. Projection: ERTS89_LAEA CRS (Lambert Azimuthal Equal Area projection, ETRS89 datum).

Despite of some gaps in taxonomic identification and nomenclature, and therefore also in knowledge of species distributions, it is still possible to present an extensive taxonomic list with identification in total of 1,476 species for the whole Baltic Sea (Figure 4). The most diverse groups are the polychaetes (275 species), crustaceans (292 species), and mollusks (308 species). Owing to the strong salinity gradient, diversity declines with decreasing salinity from the south to the brackish water areas in the north (Figure 5A). Contrarily, the number of freshwater species increases along the same gradient (Figure 5B). Especially in the more or less freshwater inshore waters (e.g., Curonian Lagoon) and in the shallow offshore waters of the northeast, the number of freshwater species (mainly insects but also oligochaetes and mollusks) increases dramatically. Naturally such an inventory is never finished, and future works will update this species list.

Because of the sharp increase in ship traffic and the anthropogenic use of offshore waters (such as pipelines for gas and oil and windmill farms), an enormous pressure on the environment could be observed in the last decades [54]. External factors, like destruction and loss of bottom habitats and introduction of alien species have changed the macrozoobenthic biodiversity of the Baltic Sea dramatically ([54], [131] and references therein).

### Fish

**Figure 6:**
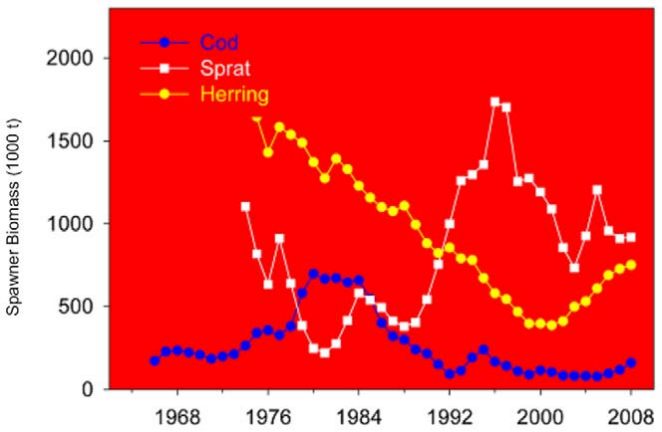
Spawner biomass of cod, herring, and sprat in the Baltic Sea (ICES management subdivisions 25–32, 25–29 (excluding the Gulf of Riga) and 22–32, respectively). Data: [137].

Overall, the Baltic Sea (including the Kattegat) fish community comprises approximately 200 species, but only about 100 established species if the Kattegat is excluded. There are around 70 species in several sub-basins in the NE Baltic, but less than 50 species in Bothnian Bay [54], [134]–[136], (Figure 2E). The biomass of fish in the Baltic is dominated by a much smaller number of species (i.e. three species: cod [*Gadus morhua*], herring [*Clupea harengus*], and sprat [*Sprattus sprattus*]). The abundance and biomass of cod, herring, and sprat (respectively in management subdivisions 25–32, 25–29, excluding the Gulf of Riga, and 22–32) have fluctuated substantially in the past 30–40 years ([137], Figure 6). Cod was at intermediate levels in the 1960s and then increased strongly in the late 1970s and early 1980s, before declining in the following 15–20 years. The increases and decreases are linked to variations in both fishing mortality and reproductive success, which itself is related to climatic-hydrographic variations and abundance of predators of cod eggs and larvae. The processes are reasonably well understood and documented [138], [139]. The recent increase is due to both lower fishing effort and mortality, and improved hydrographic conditions for reproduction. Sprat and herring biomass has also fluctuated, in part because of fluctuation in the abundance of one of their predators, cod [23], [140], [141]. Sprat and herring are key prey of larger juvenile and adult cod [23]. Additional factors that have contributed to variations in sprat and herring biomass are climatic conditions, particularly temperature [139], [142], and competition among the species for similar prey [140], [141].

Some flatfish species are commercially important and the biomass appears to be moderately high, e.g., flounder (*Platichthys flesus*) [23]. The species composition of the flatfish community has changed over time during the past century. In the early decades of the 1900s, plaice (*Pleuronectes platessa*) and dab (*Limanda limanda*) were abundant in the Bornholm Basin, but they declined in the 1950s–1970s and are rare in this area at present [139], [143]. The relatively high abundance and widespread distribution of flounder in the Baltic, but low abundance of the plaice and dab, may be partly related to differences in salinity tolerance of eggs and in reproduction [144].

Various marine fish species from the North Sea migrate from time to time into the Baltic Sea. These include whiting (*Merlangus merlangus*), European anchovy (*Engraulis encrasicolus*), mackerel (*Scomber scombrus*), grey mullet (*Liza ramada*), and thicklip mullet (*Chelon labrosus*). Owing to unfavorable environmental factors, these fish are unable to form self-sustaining populations in the Baltic. In addition, there are several noncommercial fish present in the Baltic Sea, for instance gobies (*Pomatoschistus* spp.), three-spined stickleback (*Gasterosteus aculeatus*), nine-spined stickleback (*Pungitius pungitus*), and pipefish (*Nerophis ophidion*), which are successfully adapted to low-salinity conditions prevalent in near-coastal areas and play several significant roles in the food web. However, knowledge of the spatiotemporal population dynamics of these fish is relatively poor, or in some cases even absent.

Several migratory species, such as salmon (*Salmo salar*), trout (*Salmo trutta*), eel (*Anguilla anguilla*), vimba bream (*Vimba vimba*), smelt (*Osmerus eperlanus*) are of high commercial value. Decline and or disappearance of the natural salmon stocks has been especially rapid since the late 1940s, mainly due to construction of hydroelectric power plants and river damming. However, recently some improvement is evident for the natural smolt production in the northern Baltic rivers. Sea trout populations are currently in a precarious state in the northeastern Baltic, while some improvement has been recorded in the western Baltic [145].

The most common and abundant freshwater species found in a majority of coastal areas of the Baltic Sea are perch (*Perca fluviatilis*), roach (*Rutilus rutilus*), bream (*Abramis brama*), bleak (*Alburnus alburnus*), ruffe (*Gymnocephalus cernuus*), ide (*Leuciscus idus*), pike (*Esox lucius*), and whitebream (*Blicca bjoerkna*). These fish are more abundant in areas where salinity is lower, such as in the northeastern Baltic Sea, including large gulfs and lagoons. Because of ongoing coastal eutrophication, as well as increased water temperatures, a significant increase in perch and roach abundance has been recorded in the Archipelago Sea during the past decade, while these and other species have significantly decreased, or even collapsed, in other areas. The suggested primary reason for the decline or collapse is excessive fishing pressure [26], [146].

Glacial relict species, such as fourhorned sculpin (*Triglopsis quadricornis*), sea snail (*Liparis liparis*), eelpout (*Zoarces viviparous*), and lumpsucker (*Cyclopterus lumpus*), mainly inhabit the cold-water layers in deeper areas and the northeastern Baltic Sea with favorable oxygen status. Except eelpout, these fish lack any commercial importance.Relatively limited evidence on this category of fish suggests that their abundance is negatively influenced by excessive eutrophication, contamination with toxic substances, and presence of large marine predators [135]. It is important to note that this category of fish represents a specific trophic function in the Baltic Sea; these fish are the only permanent, potentially abundant vertebrate predators in the cold-water environment in deep areas.

The salinity gradient from the North Sea east and north through the Baltic also affects biodiversity within species (i.e., intraspecific biodiversity). This is evident from recent molecular genetics studies of several fish species (cod, herring, sprat, flounder, and turbot [*Scophthalmus maximus*]). In all of these species, local genetic populations have been detected along the salinity gradient from the Skagerrak through the Danish Straits to the northern Baltic [147]–[151]. The population structuring in space is nonrandom, and most of the differences in genetic indicators of population identity occur approximately where the salinity gradient is greatest (i.e., in the Kattegat–Belt Sea–western Baltic area). These results, along with physiological, morphometric, and meristic studies (e.g., [152], [153]) show that local populations have adapted to spatial differences in environmental conditions and increased the biodiversity within individual species. Some of these adaptations have been shown to affect local reproductive success [154]. This intraspecific biodiversity is probably important for promoting resilience of these species to human impacts such as exploitation [155].

The occurrence of diseases and parasites is better known for major commercial fish species like herring, cod, flounder, and salmon [156]–[158]. In addition, long-term studies have recently resulted in a comprehensive overview of fish parasite fauna in the northeastern Baltic Sea [135]. These results reveal that the parasitofauna of the Baltic fish, and associated upper trophic levels, is substantially richer than that of the local ichthyofauna (Table 1).

This view of fish biodiversity is mainly confined to the last few decades of the twentieth century, when the Baltic Sea had already been heavily impacted by different kinds of human activities. Since the “shifting baseline” syndrome of fish abundance is quite common for marine ecosystems [159], it is therefore useful to investigate historical sources for the status of fish biodiversity before such impacts began or reached the late twentieth century intensities.

Historical archives contain significantly more information on major fish species (such as cod, herring, and salmon) than on the coastal fish. Most species exploited in the past (cod, herring, salmon, flatfishes, eel, and several freshwater fish species) are still exploited at present, but sometimes in smaller quantities [160]. This applies particularly to the sturgeon and eel populations; the sturgeon is extinct in the Baltic Sea and other studies have shown that biomass of eel is severely reduced [161]. The recovered data also show that the Baltic ecosystem was able to support modest to large cod populations, even though it was oligotrophic and contained large populations of cod predators, presumably essentially because of low fishing mortality [30], [162].

The long temporal perspective has enabled identification of how climate variability and change influences fish populations. For example, during the Atlantic Warm Period (roughly 7000 to 3900 B.C.), when temperatures resembled those likely to be typical in the late twenty-first century, cod were quite abundant near Bornholm. The high abundance is due partly to higher salinities, which were common at that time [163]. The same archaeological study showed that in the waters around Denmark (Kattegat, Skagerrak, the Belts, Bornholm), there were several warm-water fish species present during this period. At present these species have a more southerly distribution and their presence near Denmark was presumably due partly to the warmer temperatures at that time [163]. From another perspective, during the late seventeenth century (1675–96), which represents part of the coldest period of the Little Ice Age (known also as the Late Maunder Minimum), fish catches in the Gulf of Riga consisted of only three species—herring, flounder, and eelpout—while currently abundant, warm-water-preferring, and eutrophication-tolerant species were almost absent [164]. Climate may also cause substantial changes in fish phenology. During the period of substantially colder climate and severe winters in the seventeenth century, the herring fishery in the Gulf of Riga operated mostly during the summer months, probably because of the postponed migration of herring to the spawning areas close to the coast, where the fish were caught. In contrast, in the much warmer climate conditions at present, a coastal trapnet herring fishery takes place in spawning grounds a few months earlier than it did during the very cold historical times [164].

### Marine mammals

The Baltic Sea is inhabited by three species of seals. Ringed seal (*Phoca hispida*) is an Arctic species and is therefore directly dependent on quality of ice by colonizing mainly the large gulfs in the northeastern Baltic Sea (Gulf of Bothnia, Gulf of Finland, and Gulf of Riga) where ice is annually formed. The main concentrations of grey seal (*Halichoerus grypus*) are found in the northern part of the Baltic Proper. The harbor seal (*Phoca vitulina*) is present only in the southern Baltic.

Population size of the ringed seal was about 180,000–200,000 individuals in the early 1900s, but declined to about 5,000 individuals in the early 1970s [165]. Results of regular surveys (started in 1988) suggest that numbers of ringed seals have increased in the northernmost sub-basin (Bothnian Bay), but because of scarcity of data, it is not possible to derive updated abundance estimates [54]. In the beginning of the past century, the abundance of grey seal reached 90,000 individuals, but dropped to some 3,000 individuals by the end of the 1970s [165]. This population has significantly improved since then, reaching about 22,000 individuals currently [166] and was re-opened for limited national quata-based hunting since the early 2000s. Harbor seals form two distinct populations in the southern Baltic: one in the Kalmarsund area and the other in the Kattegat and Skagerrak. Both populations have faced steep declines in the first half of the twentieth century, so that their abundance was very low by the early 1970s. Multiple virus infections since the late 1980s have prevented the harbor seal population in the Kattegat and Skagerrak from fully recovering from the deep decline ([54] and references therein).

The only cetacean species reproducing in the Baltic Sea is harbor porpoise (*Phocoena phocoena*) comprising two distinct populations: one in the Baltic Proper and the other in the Kattegat and the western Baltic ([54] and references therein). Until the early twentieth century, the harbor porpoise was widely distributed and common. Population size has decreased by more than 90% during the last century and the species is currently classified as “vulnerable” ([54] and references therein). Much of the decline is presumably due to historically high levels of direct exploitation. For example, hundreds of harbor porpoises were captured annually by targeted hunting in the Little Belt, Denmark during migrations to and from the Baltic Sea [167].

### Bioinvasions

Human-mediated biological introductions have resulted in 117 alien species being recorded, about 70 of which are known to have established self-reproducing populations ([32], Tables 2 and 4). The number of known Baltic Sea aliens is about one-sixth of that in the Mediterranean Sea, and almost one-third of that on the Atlantic coast of Europe [168]. This difference is due to not only the smaller size of the Baltic Sea, but also the hostility of its brackish waters, the less intensive transoceanic shipping activity in the Baltic, and the many fewer species used for aquaculture there. On the other hand, in comparison with other bodies of brackish water in Europe (such as the Black and Caspian seas), the Baltic has the most extended salinity gradient with large β-mesohaline zone (∼300,000 km^2^, or >70% of the total area) characterized by the lowest native species richness and the highest number of alien species [169].

**Table 4:** The status of nonindigenous species by major organism groups in the Baltic Sea.

Group/Status	Established	Nonestablished	Status unknown
Phytoplankton	5	0	1
Zooplankton	5	0	3
Bottom vegetation	8	1	1
Benthic invertebrates	32	4	8
Nekto-benthic invertebrates	13	1	0
Invertebrate parasites	3	0	0
Fish	8	11	10
Birds	1	0	0
**TOTAL**	**69**	**17**	**22**

**Note:**

Includes also data from: [32].

There are very few primary introductions known in the Baltic Sea (e.g., the fishhook water flea [*Cercopagis pengoi*], zebra mussel [*Dreissena polymorpha*] and some Pacific salmonids [*Oncorhynchus* spp.]) while the Baltic has historically been and still is subject to secondary introductions from both the North Sea area and adjacent inland waters ([170] and references therein). Some brackish-tolerant species were widely distributed as forage food in the 1960–1970s, especially in former Soviet Union republics. The increase in the numbers of new introductions in the past two decades may also reflect a greater knowledge of the area [171].

Alien species are abundant and even dominant throughout the shallow benthic and fouling communities of the Baltic Sea—at present, no shallow-water habitat is entirely free of human-mediated invaders. Their number is the lowest in Bothnian Bay and the highest in the high-salinity Kattegat area (55 species, [172], Figure 2F). The most important source areas for these species have been the Atlantic coast of North America, the Ponto-Caspian region, and western European waters (Table 5). In addition, at least five species are listed as cryptogenic to the area (including the dinoflagellate *Prorocentrum minimum* and the shipworm *Teredo navalis*).

**Table 5:** Origin of the recorded alien species by selected contrasting sub-areas, and with different importance of different invasion pathways, in the Baltic Sea.

Origin	Kattegat, Belt Sea	Odra Lagoon	Vistula Lagoon	Curonian Lagoon	Gulf of Riga	Gulf of Finland	Bothnian Bay
Africa	1	0	0	0	0	1	0
Arctic waters	0	0	0	0	0	0	1
Asia (inland waters)	0	0	5	3	7	7	1
China Seas	3	1	1	1	1	1	1
Indian Ocean	1	0	0	0	0	0	1
Indo-Pacific	5	2	1	1	0	1	0
Japan Sea	4	0	0	0	0	0	0
North America	21	6	6	6	5	5	11
Pacific	10	1	3	2	2	4	3
Ponto-Caspian	2	11	15	16	10	21	5
West Europe (Atlantic coast)	1	0	2	0	0	1	1
West Europe (inland)	1	1	0	0	1	0	0
Unknown	3	0	0	1	0	0	0
**TOTAL**	**52**	**22**	**33**	**30**	**26**	**41**	**24**

**Note:**

Includes also data from: [32].

Ship traffic remains the most important pathway of introduction in the Baltic Sea. The sea and its drainage area are connected to the Ponto-Caspian brackish seas (Black, Azov, and Caspian seas) by rivers and canals. Some 250 rivers discharge fresh water into the Baltic from a drainage area that is four times greater than its sea surface area. Consequently, every non-native species released into the wild somewhere in the drainage basin can be transported to the sea or its most diluted coastal areas. In the peripheral parts of the drainage area of the Baltic, these species consist mainly of the Ponto-Caspian biota, whereas the northwest European river mouths host a number of marine and brackish-water aliens native to other seas [170].

Potentially toxic dinoflagellate, *P. minimum*, invaded the Baltic Sea (exept the Gulf of Bothnia) in the last two decades of the twentieth century causing strong algal blooms in coastal areas [173]. A bloom of the toxic raphidophyte *Chattonella marina* (now *Pseudochattonella farcimen*) occurred in the Gullmar fjord, Sweden, in spring 2001 [174].

Alien species have no direct value as food resources in the Baltic, as none of them supports commercial fisheries and invertebrates are not harvested for food because of their small size. Some planktonic invaders (e.g., the fishhook water flea, *C. pengoi*) have a high value as a food source for commercially harvested fish, such as the Baltic herring (e.g., [175]). However, on the basis of existing knowledge, approximately 30 nonindigenous species (i.e., less than 30% of all introduced species) can be classified as nuisance organisms in the Baltic; only 9 of them have caused measurable damage. These are 4 Ponto-Caspian species (*C. pengoi*, *C. caspia*, *D. polymorpha* and *Neogobius melanostomus*), three North-American species (*Balanus improvisus*, *Gammarus tigrinus* and the American mink *Mustela vison*), the Japanese swim-bladder nematode *Anguillicola crassus* and the “shipworm” mollusk *T. navalis*, believed to be of Indo-Pacific origin. The clogging of reels and fouling of nets makes *C. pengoi* a potential nuisance species, this may have caused substantial economic loss in fisheries [176]. The cryptogenic shipworm *T. navalis*, now fully established in the southwestern Baltic, has caused remarkable damage to submerged wooden installations (K. Hoppe unpublished) and marine archaeological objects [170]. The influence of the most recent and potentially harmful invader—the alien ctenophore *Mnemiopsis leidyi*—on the pelagic food web of the Baltic Sea seems to be spatially restricted to the southern Baltic Sea [177], where amongst other prey (e.g., copepods, *nauplii* of the alien cirriped *B. improvisus* and larvae of jellyfish *Aurelia aurita*) cannibalism on larval *M*. *leidyi* was observed [178].

## Discussion

The most recent overview on threats to the biodiversity of the Baltic Sea includes the lists of the following 10 major categories: fisheries, maritime activities (including shipping), physical damage and disturbance, recreational activities, eutrophication, hazardous substances, alien species, noise pollution, hunting, and climate change [54]. Overfishing, eutrophication, and drastic decline of marine mammals have been the most prominent changes in the Baltic Sea during the twentieth century [167]. While remarkable increase in fishing mortality for some species (eastern Baltic cod stock) has been evident since the mid-1940s [162], known measures to protect some migratory species (e.g., sturgeon) during the spawning season originated in the southern Baltic at least as early as the sixteenth century [11]. The impact of fishing on Baltic fish stocks certainly intensified with implementation of trawling, which took place in the 1920s, and allowed fishing to move farther offshore [179]. The first signs of eutrophication became evident in the mid-1950s, and the eutrophication status in most areas of the Baltic Sea is currently poor or bad, excluding the Gulf of Bothnia, of which subareas are predominantly good or moderate. In the Baltic Sea, eutrophication has led to shrinkage of distribution area and population declines of species preferring clear and oxygen-rich water, impoverishment of species diversity, increased bioproductivity, and intensification of potentially toxic cyanobacterial blooms [180]. It has been suggested that the decline of seal populations by about one order of magnitude results from a combination of excessive hunting initially, followed by toxic pollution [165].

All the other factors affecting the Baltic biodiversity are of relatively recent concern and have localized impact, or information on their impact is poorly documented (because the stressor is relatively recent). Although the first human-mediated introduction of species in the Baltic Sea occurred in the eleventh to twelfth century, species invasions have become a problem during the past two decades, especially with intensified invasion of alien species from the Ponto-Caspian region. In contrast with many other seas, invasion of alien species has increased both species and functional diversity of the Baltic Sea [181] and currently, both coastal and offshore areas are affected by alien species [170]. In addition to bioinvasions-related stress, several other human activities are increasing as well. These include maritime transport (with increased risk of oil spills), extraction and disposal activities, a variety of technical installations in coastal areas and on the seabed (including energy pipelines), and recreational activities. It has been recently shown elsewhere that impacts due to fishing increase the vulnerability of exploited populations, and consequently the ecosystems of which they are a part, to other perturbations such as natural and human-induced climate variations, eutrophication, and habitat changes [182], [183]. Thus, the likelihood that the Baltic Sea biodiversity is further affected by a variety of human activities is increasing.

Sub-regionally balanced and representative species diversity analysis was possible to carry out for six organism groups (Figure 2). The steep temperature and salinity gradients from Kattegat to NE Baltic Sea significantly influence both species composition as well as diversity with the number of marine taxa increasing and that of limnetic species decreasing along with increase in salinity gradient [184]. The current study has evidenced that in case of some organism groups, e.g. benthos and fish, overall species richness is substantially higher at higher salinities. However, through providing suitable habitats for both marine and limnic species, some distinct sub-basins (e.g., Gulf of Finland) may host comparably similar or even higher number of species than recorded at high salinities in Kattegat. Although the absolutely similar sampling effort for marine biodiversity studies can never be achieved (and this is mostly likely the case also here), we hereby argue that, there should be no major problems in making sub-regional comparisons of the six organism groups considered (Figure 2), and the basic conclusions drawn from these sub-regional comparisons. Importantly, the information and data utilized include also the expert knowledge through the regional networks (including HELCOM), making thereby the present paper as the first comprehensive inventory for the Baltic biodiversity ever published. Thus, the major conclusions drawn here can not be considered as a function of biased sampling.

HELCOM started to establish a system of marine and coastal Baltic Sea Protected Areas (BSPAs) in 1994, with the overall aim of contributing to the protection of the entire ecosystem, including all its components and functions, and not just certain species or habitats. All contracting parties to the Helsinki Convention contributed by identifying and nominating an initial suite of 62 sites. The protected areas should therefore be well distributed across the Baltic Sea area and its different subsystems to include all species, habitats, and ecosystems [180]. Overall, the currently existing BSPA network can be considered adequate for the size of most sites, whereas the geographical coverage and distribution are inadequate, because the network covers less than 10% of the entire Baltic Sea. Especially poorly represented are offshore and deepwater areas [54].

The lists of threatened or declining species and biotopes or habitats of the Baltic Sea area contain 61 species [53]. All these are in urgent need of protective measures. The need for their protection is also highlighted in the HELCOM BSAP [49]. While bird and mammal species are well represented in the BSPAs, the other taxa are weakly represented: about half of the threatened or declining species (29 of 61) are not included in the current BSPA network [54].

The Baltic Sea is one of the most intensively studied regional seas in the world; some continuous datasets go back to the early 1950s. Despite this, substantial gaps in knowledge still occur and some of the pressing issues are discussed below. So far, diversity of the biota of the Baltic Sea has been routinely described for dominant species of certain groups or size fractions that are identified and counted for monitoring purposes. Smaller organisms, like unicellular and colony-forming picocyanobacteria, which could make up a substantial part of phytoplankton biomass [42] or microzooplankton are certainly poorly studied. For instance, present geographical coverage of the Baltic Sea is still incomplete for Protista, Rotifera, and Brachiopoda [185] which, amongst others, hampers performing of sub-regional species richness evaluations (see also Figure 2). It should be also mentioned that the recent integrated thematic assessment on biodiversity and nature conservation of the Baltic Sea [54] lacks information for groups such as bacterioplankton and meiobenthos, but also for diseases and parasites of marine organisms. All these organism groups suffer under insufficient taxonomic expertise and identification guides in the Baltic Sea, which have resulted in unsatisfactory information on their taxonomic composition and population characteristics as well as their biology and ecology. Further, there is a need for additional geographically representative field data on bottom vegetation to provide good coverage of a variety of different environments. Finally, distribution and abundance or biomass of noncommercial fish, including sticklebacks (*G. aculeatus* and *P. pungitius*), gobies (*Pomatoschistus* spp.), fourhorned sculpin (*T. quadricornis*), sea snail (*L. liparis*), and eelpout (*Z. viviparus*), is also scarce, despite the fact that these species may play significant roles in the ecosystem. These gaps result from a combination of lack of expertise and of relevant monitoring and research programs because of a shortage of financial resources.

Nowadays, it is a common problem worldwide that professional taxonomists with extensive knowledge of the systematics of different groups of aquatic invertebrates are becoming extinct [186]. For the Baltic Sea region, it is exceptionally important to incorporate biodiversity research into routine monitoring programs, coordinate Baltic Sea faunistic inventory projects, increase taxonomic training of professional staff in hydrobiological laboratories, recruit younger generation and store results in joint databases to harmonize methods and improve skills necessary for taxonomic identification (e.g., [17]). The training courses for taxonomic identification (by organism groups) are essential for acquiring and maintaining the quality assurance of the laboratories participating in the joint international monitoring programs in the Baltic Sea region.

Application of various molecular techniques is an increasingly important tool in marine biodiversity studies. Several of these techniques have already been applied to study genetic diversity at different trophic levels of the Baltic Sea. For instance, case studies are available to show that two genotypes of the cyanobacteria *Nodularia* and one genetically valid species of *Anabaena* exist in the Baltic Sea [187], [188]. In addition, molecular tools have enabled to identify the donor-region of the alien polychaete *Marenzelleria* spp. [189] and resolve taxonomic identification of an alien ctenophore species [177]. Also, existence of spatial subpopulations of the harbour porpoise was confirmed by genetic methods [190].

By housing unique genes, genotypes and populations, the Baltic Sea is a vulnerable, but exceedingly valuable genetic resource [191]. However, sustainable management of this genetic resource still remains a challenge. For instance, sufficient data to provide basic information on genetic structure and genetic units for biologically sustainable use are available for only six commercially exploited fish species, but the current management practices do not sufficiently consider even these data [192]. To make a better use of accumulating genetic data and provide a bridge between landscape ecology and population genetics, a new discipline called ‘landscape genetics’ has been developed. Amongst others, landscape genetics may provide important new information not only on the selection and local adaptations in marine environments, but also enable identify management units which better correspond to barriers of gene flow [193].

## Supporting Information

Text S1 Reference list of manuals and identification guides.
(0.07 MB DOC)
