# Supplementary material for: Status of Biodiversity in the Baltic Sea
Source: PLoS One. 2010 Sep 1;5(9):e12467. doi: 10.1371/journal.pone.0012467 (PMC2931693; doi:10.1371/journal.pone.0012467)
Supplement: Text S1 — Reference list of manuals and identification guides. (0.07 MB DOC) [file pone.0012467.s001.doc]

**Text S1. Reference list of Manuals and Identification Guides**

**Cyanobacteria and phytoplankton**

1. HELCOM (1988) Guidelines for the Baltic Monitoring Programme for the third stage: Part D. Biological Determinands. Baltic Sea Environ. Proc. 27D. Helsinki: HELCOM.
2. HELCOM COMBINE manual. [www.helcom.fi/groups/monas/CombineManual/AnnexesC/en_GB/annex6](http://www.helcom.fi/groups/monas/CombineManual/AnnexesC/en_GB/annex6), Accessed 30 October 2009.
3. Hoppenrath M, Elbrächter M, Drebes G (2009) Marine Phytoplankton. Selected microphytoplankton from the North Sea around Helgoland and Sylt. Koeltz Scientific Books.
4. Hällfors G (2004) Checklist of Baltic Sea phytoplankton species (including some heterotrophic protistan groups). Balt Sea Environ Proc 95: 1−208.
5. Joosten T (2006) Flora of the bluegreen algae of the Netherlands. Volume 1. KNNV Publishing.
6. Komarek J, Anagnostidis K (1999) Cyanoprocaryota 1. Teil: Chroococcales. Süßwasserflora von Mitteleuropa 19/1: 1−548. Gustav Fischer Verlag, Jena.
7. Komarek J, Anagnostidis K (2005) Cyanoprocaryota 2. Teil: Oscillatoriales. Süßwasserflora von Mitteleuropa 19/2:1−759. Elsevier. Spektrum Akademischer Verlag.
8. Pankow H (1990) Ostsee-Algenflora. Jena: Gustav Fischer Verlag. 648 p.
9. Thomsen HA, editor (1992) Plankton i de inre danske farvande. Analyse af forekomsten af alger og heterotrofe protister (ekskl. ciliater) i Kattegat. Havforskning fra Miljøstyrelsen 11:1−331.
10. Tikkanen T, Willén T (1992) Växtplanktonflora. In: Tomas C, editor. Naturvårdsverket Förlag. Academic Press. 280 p.

**Phytobenthos**

1. Brodie JA, Irvine LM (2003) Seaweeds of the British Isles. Volume 1 Rhodophyta, Part 3B Bangiophycidae. The Natural History Museum, London. 167 p.
2. Burrows EM (1991) Seaweeds of the British Isles. Volume 2 Chlorophyta. The Natural History Museum, London. 238 p.
3. Dixon PS, Irvine LM (1995) Seaweeds of the British Isles. Volume 1 Rhodophyta. Part 1 Introduction, Nemaliales, Gigartinales. The Natural History Museum, London. 252 p.
4. Fletcher RL (1987) Seaweeds of the British Isles, 3: Fucophyceae (Phaeophyceae), Part 1. The Natural History Museum, London. 359 p.
5. Langangen A (2007) Charophytes of the Nordic countries. Saeculum, Oslo. 101 p.
6. Leinikki J, Backer H, Oulasvirta P, Leinikki S, Ruuskanen A (2004) Aaltojen alla – Itämeren vedenalaisen luonnon opas. Gummerus Kirjapaino Oy, Jyväskylä, 144 p.
7. Maggs CA, Hommersand MH (1993) Seaweeds of the British Isles. Volume 1 Rhodophyta. Part 3A Ceramiales. The Natural History Museum, London. 444 p.
8. Nielsen R, Kristiansen A, Mathiesen L, Mathiesen H (1995). Distribution index of the benthic macroalgae of the Baltic Sea area. In: Acta Botanica Fennica 155. 55 p.
9. Pankow H (1990) Ostsee-Algenflora. Jena: Gustav Fischer Verlag. 648 p.
10. Schubert H, Blindow I, editors (2003) Charophytes of the Baltic Sea. BMB Publ. No. 19. Koeltz scientific: Königstein. 332 p.
11. Tolstoy A, Österlund K (2003) Alger vid Sveriges östersjökust – en fotoflora. Art Databanken, Uppsala. 282 p.

**Zooplankton**

1. Alekseev VR (2002) Copepoda. In: Fernando CH, editor. A Guide to Tropical Freshwater Zooplankton. Leiden: Backhuys Publishers. pp. 123−187.
2. Czaika SC (1982) Identification of nauplii N1-N6 and copepodids CI-CIV of the Great Lakes calanoid and cyclopoid copepods (Calanoida, Cyclopoida, Copepoda). J Great Lakes Res 8(3): 439−469.
3. Dumont HJ, Negrea SV (2002) Introduction to the class Branchiopoda, Vol. 19. In: Dumont HJF, editor. Guides to the identification of the microinvertebrates of the continental waters of the world. Leiden: Backhuys Publishers. 398 p.
4. Dussart BH, Defaye D (2001) Introduction to the Copepoda, Vol. 16. In: Dumont HJF, editor. Guides to the identification of the microinvertebrates of the continental waters of the world. Second edition. Leiden: Backhuys Publishers. 344 p.
5. Einsle U (1993) Crustacea, Copepoda: Calanoida und Cyclopoida; Susswasserfauna von Mitteleuropa. Stuttgart, Jena, New York: Gustav Fischer Verlag. 208 p.
6. Einsle U (1996) Copepoda: Cyclopoida. Genera Cyclops, Megacyclops, Acanthocyclops, Vol. 10. In: Dumont HJF, editor. Guides to the identification of the microinvertebrates of the continental waters of the world. The Hague: SPB Academic Publishing. 83 p.
7. Flössner D (2000) Die Haplopoda und Cladocera Mitteleuropas. Leiden: Backhuys Publishers. 428 p.
8. Foissner W, Berger H (1996) A user-friendly guide to the ciliates (Protozoa, Ciliophora) commonly used by hydrobiologists as bioindicators in rivers, lakes, and waste waters, with notes on their ecology. Freshwat Biol 35: 375−482.
9. Gerlach S (2000) Checkliste der Fauna der Kieler Bucht und eine Bibliographie zur Biologie und Ökologie der Kieler Bucht. Die Biodiversität in der deutschen Nord- und Ostsee. Bundesanstalt für Gewässerkunde Koblenz. 376 p.
10. Hayward PJ, Ryland JS, editors (2005) Handbook of the Marine Fauna of North-West Europe. New York: Oxford University Press Inc. 800 p.
11. HELCOM (2005) Manual for marine monitoring in the COMBINE programme of HELCOM, Part C. [www.helcom.fi/groups/monas/CombineManual/PartC/en_GB/main/](http://www.helcom.fi/groups/monas/CombineManual/PartC/en_GB/main/), Accessed 7 November 2009.
12. Huys R, Baxshall G (1991) Copepod evolution. London: The Ray Soc. 468 p.
13. ICES (2000) ICES Zooplankton Methodology Manual. San Diego, San Francisco, New York, Boston, London, Sydney, Tokyo: Academic Press. 684 p.
14. Kahl A (1930-1935) Urtiere oder Protozoa; 1: Wimpertiere oder Ciliata (Infusoria), I-IV. Jena: Gustav Fischer Verlag.
15. Kiefer F (1978) Das Zooplankton der Binnengewässer. Freilebende Copepoda. Die Binnengewässer, Stuttgart, 26: 1−343.
16. Korinek V (2002) Cladocera. In: Fernando CH, editor. A guide to tropical freshwater zooplankton. Leiden: Backhuys Publishers. pp. 69−122.
17. Koste W (1978) Rotatoria. Die Rädertiere Mitteleuropas. Bd 1-2. Gebrüder Borntraeger, Berlin, Stuttgart.
18. Kutikova LA (1970) Rotifers of the USSR. Fauna USSR 104. Leningrad: Akad. Nauk USSR. 744 p.
19. Larink O, Westheide W (2006) Coastal plankton. Photo guide for European seas. München: Verlag Dr. Friedrich Pfeil. 144 p.
20. Manuilova EF (1964) Cladocera of the USSR fauna. Moscow-Leningrad. 326 p.
21. Mordukhai-Boltovskoi PhD, Rivier IK (1987) Predatory Cladocera (Onychopoda: Podonidae, Polyphemidae, Cercopagidae, and Leptodoridae) of the world fauna. Leningrad: Nauka. 184 p.
22. Smirnov NN (1996) Cladocera: The Chydorinae and Sayciinae (Chydoridae) of the World, Vol. 11. In: Dumont HJF, editor. Guides to the identification of the microinvertebrates of the continental waters of the world. The Hague: SPB Academic Publishing. 97 p.
23. Telesh I, Heerkloss R (2002) Atlas of Estuarine Zooplankton of the Southern and Eastern Baltic Sea. Part I: Rotifera. Hamburg: Verlag Dr. Kovač. 89 p.
24. Telesh I, Heerkloss R (2004) Atlas of estuarine zooplankton of the southern and eastern Baltic Sea. Part II: Crustacea. Hamburg: Verlag Dr. Kovač. 118 p.
25. Telesh I, Postel L, Heerkloss R, Mironova E, Skarlato S (2008) Zooplankton of the open Baltic Sea: Atlas. BMB Publication 20. Meereswiss. Ber. Warnemünde 73: 1–251. [www.io-warnemuende.de/marine-science-reports.html](http://www.io-warnemuende.de/marine-science-reports.html), Accessed 7 November 2009.
26. Telesh I, Postel L, Heerkloss R, Mironova E, Skarlato S (2009) Zooplankton of the open Baltic Sea: Extended atlas. BMB Publication 21. Meereswiss. Ber. Warnemünde 76: 1–290. [www.io-warnemuende.de/marine-science-reports.html](http://www.io-warnemuende.de/marine-science-reports.html), Accessed 7 November 2009.
27. Westheide W, Rieger R (1996) Spezielle Zoologie, Teil 1: Einzeller und Wirbellose Tiere. Gustav Fischer Verlag, Stuttgart, Jena, New York. 909 p.

**Meiozoobenthos**

1. Bartsch I (1972) Ein Beitrag zur Systematik, Biologie und Okologie der Halacaridae (Acari) aus dem Litoral der Nord- und Ostsee. I. Systematik und Biologie. Abh Verh Naturwiss Ver Hamburg (NF) 16: 155-230.
2. Brinkhurst RO (1982) Oligochaeta. In: Parker SP, editor. Synopsis and classification of living organisms. New York: McGraw Hill. pp. 50-61.
3. Elofson O (1941) Zur Kenntnis der marinen Ostracoden Schwedens. Zool Bidr Uppsala 19: 217-534.
4. Erseus C (1980) Specific and generic criteria in marine Oligochaeta, with special emphasis on Tubificidae. In: Brinkhurst RO, Cook DG, editors. Aquatic Oligochaete Biology: Plenum, London pp. 9-24.
5. Hartmann G (1963) Zur Phylogenie und Systematik der Ostracoden. Zeitschr Zool Syst Evolutionforsch 1: 1-154.
6. Hartmann G (1966) Ostracoda. In: Dr. H.G.Bronns Klassen und Ordnungen des Tierreichs. 2 (4): 1-216.
7. Higgins RP, Thiel H, editors (1988) A manual for the study of meiofauna. Washington, D.C.: Smithsonian Institution Press. 488 p.
8. Hummon WD, Todaro MA, Evans WA (2005) Video database for described species of marine Gastrotricha. Meiofauna Marina: 14: 23-26.
9. Huys R, Gee JM, Moore CG, Hamond R (1996) Marine and brackish water harpacticoid copepods: Part I. Keys and notes for identification of the species. Synopses of the British Fauna, 51. 352 p.
10. Karling TG (1974) Turbellarian fauna of the Baltic Proper. Identification, ecology and biogeography. Fauna Fennica 27: 1-101.
11. Kisielewski J (1975) Brzuchorzęski (Gastrotricha) psammonowe Polskiego Bałtyku. Bad Fizj Polski Zach. 28: 7-40 (in Polish).
12. Kisielewski J (1997) Brzuchorzęski (Gastrotricha). Fauna Słodkowodna Polski 31: 1-157 (in Polish).
13. Lang K (1948) Monographie der Harpacticiden. Lund: Hakan Ohlsson. 1682 p.
14. Platt HM, Warwick RM (1983) Free-living Marine Nematodes. Part I. British Enoplids. Synopses of the British Fauna (New Series), 28. 307 p.
15. Platt HM, Warwick RM (1988) Free-living Marine Nematodes. Part II. British Chromadorids. Synopses of the British Fauna (New Series), 38. 502 p.
16. Radwan S, Bielańska-Grajner I, Ejsmont-Karabin J (2004) Wrotki (Rotifera). Fauna Słodkowodna Polski, 32.
17. Rahm G (1928) Tardigrada. In: Grimpe G, Wagler E, editors. Die Tierwelt der Nord- und Ostsee. Teil XI: b1-b56.
18. Remane A (1927). Gastrotricha. In: Grimpe G, Wagler E, editors. Die Tierwelt der Nord- und Ostsee. Teil VII: d1-d56.
19. Remane A (1927) *Halammohydra*, ein eigenartiges Hydrozoon der Nord- und Ostsee. Zeitschrift fuer Morphologie und Oekologie der Tiere 7: 643-677.
20. Remane A (1928) Kinorhyncha. In: Grimpe G, Wagler E, editors. Die Tierwelt der Nord- und Ostsee. Teil VII: 57-84.
21. Thane-Fenchel A (1968) A simple key to the genera of marine and brackish-water rotifers. Ophelia 5: 299-311.
22. Todaro MA, Hummon WD (2008) An overview and a dichotomous key to genera of the phylum Gastrotricha. Meiofauna Marina 16: 3-20.
23. Viets K (1927) Halacaridae. In: Grimpe G, Wagler E, editors. Die Tierwelt der Nord- und Ostsee. Teil XI: c1-c72.
24. Warwick RM, Platt HM, Somerfield PJ (1998) Free-living Marine Nematodes. Part III. Monhysterids. In: Synopses of the British Fauna (New Series) 53. 296 p.
25. Wells JBJ (1976-1985). Keys to Aid in the Identification of Marine Harpacticoid Copepods. University of Aberdeen, and Amendmends: Bulletins in Zoology Publications from Victoria University of Wellington.

**Macrozoobenthos**

1. Bick A, Gosselck F (1985) Arbeitsschlüssel zur Bestimmung der Polychaeten der Ostsee. Mitteilungen des Zoologischen Museums Berlin 61: 171−272.
2. Graham A (1988) Molluscs: prosobranch and pyramidellid gastropods. Keys and Notes for the Identification of the Species. Second edition. Synopsis of the British Fauna, New Series 2. 662 p.
3. Hartmann-Schröder G (1996) Annelida, Borstenwürmer, Polychaeta. (2. Auflage). In: Dahl F, editor. Die Tierwelt Deutschlands und der angrenzenden Meeresteile nach ihren Merkmalen und ihrer Lebensweise. Teil 58. Jena: Gustav Fischer Verlag. 648 p.
4. Hayward PJ, Ryland JS (1990) The marine fauna of the British Isles and north-west Europe. Vol. 1: Introduction and protozoans to arthropods. Vol. 2: Molluscs to chordates. Oxford: Clarendon Press. 996 p.
5. Jagnow B, Gosselck F (1987) Bestimmungsschlüssel für die Gehäuseschnecken und Muscheln der Ostsee. Mitteilungen des Zoologischen Museums Berlin 63: 191−268.
6. Köhn J, Gosselck F (1989) Bestimmungsschlüssel der Malakostraken der Ostsee. Mitteilungen des Zoologischen Museums Berlin 65: 3−114.
7. Lincoln RJ (1979) British marine amphipoda: Gammaridea. London: British Museum of Natural History. 658 p.
8. Thompson TE (1988) Molluscs: Benthic opisthobranchs (Mollusca: Gastropoda). Keys and notes for the identification of the species. Second edition. Synopses of the British Fauna, New Series 8: 356 p.

**Fish parasites**

1. Bauer ON, editor (1984) Identification guide of freshwater fish parasites of SSSR. Leningrad: Nauka (in Russian).
2. Bruno DW, Nowak B, Elliott DG (2006) Guide to the identification of fish protozoan and metazoan parasites in stained tissue sections. Diseases of aquatic organisms 70: 1-36.
3. Bucke D, Vethaak AD. Lang T, Mellergaard S (1996) Common diseases and parasites of fish in the North Atlantic: Training guide for identification. ICES Techniques in Marine Environmental Sciences 19: 27 p.
4. Bychovskaya-Pavlovskaya IE (1985) Parasites of fishes: Guide for investigation. 118 p (in Russian).
5. Fagerholm HP (1992) Systematic implications of male caudal morphology in ascaridoid nematode parasites. Syst. Parasitol. 19: 215-228.
6. Grabda J (1991) Marine fish parasitology: An outline. Warszawa: PWN 306 p.
7. ICES (1989) Methodology of fish disease surveys. ICES Cooperative Research Report, 166. 33 p.
8. ICES (2006) Report of the ICES/BSRP Sea-going Workshop on Fish Disease Monitoring in the Baltic Sea (WKFDM), 5–12 December 2005. ICES CM 2006/BCC:02. 89 p.
9. Kinne O, editor (1984-85). Diseases of marine animals. Vol. IV. Hamburg: Biologische Anstalt Helgoland. 884 p.
10. Moravec F (1994) Parasitic nematodes of freshwater fishes of Europe. Dordrecht: Kluwer Academic Publishers.

**Fish**

1. Berg LS (1948) Freshwater fishes of the USSR and adjacent countries 1. Moscow-Leningrad: AN SSSR. 466 p.
2. Berg LS (1949) Freshwater fishes of the USSR and adjacent countries 2,3. Moscow-Leningrad: AN SSSR. pp 467-1382.
3. European Commission (EC) (2008) Commission Decision adopting a Multiannual Community Programme of Commission Regulation (EC) No 199/2008 establishing a Community framework for the collection, managament and use of data in the fisheries sector and support for scientific advice regarding the common fisheries policy. Official Journal of the European Union L346: 37−78.
4. HELCOM (2006) Assessment of Coastal Fish in the Baltic Sea. Balt. Sea Environ Proc No 103 A. Helsinki: HELCOM. 26 p.
5. ICES (2009) Report of the ICES working group on Baltic international fish survey (WGBIFS). ICES CM 2009/LRC:05. Copenhagen: ICES. 75 p.
6. Muus BJ, Nielsen JG (1999) Sea fish. Scandinavian Fishing Year Book, Hedehusene, Denmark. 340 p.
7. Ojaveer E, Pihu E, Saat T, editors (2003) Fishes of Estonia. Tallinn: Estonian Academy Publishers. 416 p.
8. Thoresson G (1993) Guidelines for coastal monitoring – Fishery biology. Kustrapport 1: 1-35.
9. Wheeler A (1978) Key to the Fishes of Northern Europe. London: Frederick Warne & Co. Ltd. 380 p.

**Mammals and birds**

1. Aulagnier S, Haffner P, Mitchell-Jones AJ, Moutou F, Zima J (2009). Mammals of Europe, North Africa and the Middle East. London: Christoper Helm Publishers.
2. Cramp S, Snow D, Perrins CM (1997). The Birds of the Western Palearctic, Concise Edition. Oxford: Oxford University Press.
3. Delany S, Reyes C, Hubert E, Pihl S, Rees E, Haanstra L, Strien A (1999). Results from the International Waterbird Census in the Western Palearctic and Southwest Asia 1995 and 1996. Wetlands International Publication 54. 178 p.
4. Hagemeijer W, Blair M (1997). The EBCC atlas of European breeding birds. Their distribution and abundance. London: T. & A.D. Poyser Ltd. 960 p.
5. Jonsson L (2006) Birds of Europe with North Africa and the Middle East. London: Christoper Helm Publications. 559 p.
6. MacDonald DW, Barrett P (1993) Collins field guide to mammals of Britain and Europe. Harper Collins Publishers, 312 p.
